# Neural speech tracking and auditory attention decoding in everyday life

**DOI:** 10.3389/fnhum.2024.1483024

**Published:** 2024-11-13

**Authors:** Lisa Straetmans, Kamil Adiloglu, Stefan Debener

**Affiliations:** ^1^Department of Psychology, Neuropsychology Lab, University of Oldenburg, Oldenburg, Germany; ^2^Hörzentrum Oldenburg gGmbH, Oldenburg, Germany; ^3^Sonova Consumer Hearing GmbH, Wedemark, Germany; ^4^Research Center Neurosensory Science, University of Oldenburg, Oldenburg, Germany; ^5^Cluster of Excellence Hearing4all, University of Oldenburg, Oldenburg, Germany

**Keywords:** mobile EEG, speech envelope tracking, auditory attention decoding, distraction, movement

## Abstract

**Introduction:**

In our complex world, the auditory system plays a crucial role in perceiving and processing our environment. Humans are able to segment and stream concurrent auditory objects, allowing them to focus on specific sounds, such as speech, and suppress irrelevant auditory objects. The attentional enhancement or suppression of sound processing is evident in neural data through a phenomenon called neural speech tracking. Previous studies have identified correlates of neural speech tracking in electroencephalography (EEG) data, but EEG measures are susceptible to motion artefacts, and the association between neural data and auditory objects is vulnerable to distraction.

**Methods:**

The current study investigated EEG-based auditory attention decoding in realistic everyday scenarios. N=20 participants were exposed to the sound of a busy cafeteria or walked along busy and quiet streets while listening to one or two simultaneous speech streams. We also investigated the robustness of neural speech tracking estimates within subjects. Linear decoding models were used to determine the magnitude of neural speech tracking.

**Results:**

The results confirmed that neural speech tracking was strongest in single speaker scenarios. In dual speaker conditions, there was significantly stronger neural speech tracking for the attended speaker compared to the ignored speaker, even in complex environments such as a busy cafeteria or outdoor settings.

**Discussion:**

In conclusion, EEG-based attention decoding is feasible in highly complex and realistic everyday conditions while humans behave naturally.

## 1 Introduction

We regularly face complex listening challenges when we are on a busy train, walking through a crowded city, or having a social gathering with friends. In such situations, people are constantly exposed to a number of different, overlapping sound sources such as speech, music, or traffic noise. Auditory scene analysis requires separating and identifying different auditory objects, suppressing irrelevant information, and advanced processing of relevant information (Kaya and Elhilali, 2017). The segmentation and streaming of different auditory objects can be very demanding and may require a considerable amount of attentional resources (Herrmann and Johnsrude, 2020). Many hearing-impaired individuals have difficulty separating auditory objects from each other, making multi-talker settings particularly challenging for this population (Shinn-Cunningham and Best, 2008). State-of-the-art hearing aids may not provide benefits in such complex settings (Lesica, 2018). It has been proposed that brain electrical activity, as captured by electroencephalography (EEG), could be used to assist the auditory system in such complex tasks, for example, by continuously adjusting hearing aid configurations to enhance the auditory objects to be attended (Geirnaert et al., 2021b). Although considerable progress has been made, most of the work on combining hearing aids with EEG-based attention decoding is at a low technology readiness level.

Low-frequency features of speech streams can be associated with simultaneously recorded neural data, a phenomenon that may be best described as neural speech tracking. A common finding is that the association between EEG and audio signals is modulated by attention (Ding and Simon, 2012a, 2012b; O’Sullivan et al., 2015). Accordingly, evaluating the correlation between neural data and speech envelopes allows for brain data-driven auditory attention decoding, i.e., the identification of an attended speaker in a multi-speaker environment from the EEG signal (e.g., Jaeger et al., 2020; Mirkovic et al., 2015). However, most studies in this area have implemented rather simple, stationary listening configurations, where listeners and sound sources did not move and the overall listening demands did not fluctuate over time. Such scenarios do not capture very well the highly dynamic listening demands we face in real life, where sound objects move and listeners move as well.

While EEG signals are susceptible to motion artifacts (e.g., Jacobsen et al., 2020), the neural correlates of selective auditory attention have been successfully recorded with mobile EEG systems in freely outdoors walking individuals (De Vos et al., 2014; Debener et al., 2012; Reiser et al., 2020). In stationary settings, the association between audio and EEG has been found to be modulated by visual or auditory distractions, which may be common in natural environments (Holtze et al., 2021; Kaya and Elhilali, 2017). Indeed, transient auditory distractors briefly disrupt auditory attention decoding (Holtze et al., 2021; Straetmans et al., 2021). However, continuous background noise could also be stimulating and facilitate the allocation of auditory attention (Herrmann and Johnsrude, 2020; Shinn-Cunningham and Best, 2008). In any case, cognitive resource limits may be reached earlier when listeners experience frequent distraction, high task demands, fatigue, or overstimulation (Herrmann and Johnsrude, 2020).

When a listener walks with another person the task at hand is not only to carry on a conversation but also to control one’s own gait and synchronize gait patterns (e.g., Scanlon et al., 2022). In non-stationary conditions, motor demands can be considered a secondary task to cognitive processing, which may place a greater burden on capacity-limited resources available for a cognitive task such as attentive listening (Al-Yahya et al., 2011; Leone et al., 2017). However, a listener may also disengage if the listening task at hand is not sufficiently relevant or inspiring (Herrmann and Johnsrude, 2020).

We recently reported the first evidence for successful EEG-based auditory attention decoding in mobile scenarios (Straetmans et al., 2021). Participants either sat in a chair or walked freely around a large, quiet, and empty university cafeteria while attending to one out of two simultaneously presented speech streams. Results confirmed above chance auditory attention decoding during free walking, even when salient auditory events occasionally distracted listeners from the primary task (Straetmans et al., 2021). The current study aimed to replicate the main finding of successful EEG auditory attention decoding in freely walking listeners. Our primary goal was to test whether auditory attention decoding is possible in a complex real-world environment, during natural behavior. Specifically, we asked whether EEG-based auditory attention decoding is possible when listeners are immersed in a natural, challenging listening environment, i.e., a typical city street soundscape or a busy cafeteria scene. For this purpose, we recorded data in two listening contexts, hereafter referred to as Lab and Beyond-the-lab conditions. The first recording context was a Lab condition and served as a baseline (hereafter referred to as Lab 1). Participants were seated in the center of a loudspeaker ring while being presented with single and dual speech narratives, which were presented with or without background noise. In the background noise conditions, the speech streams were embedded in a busy cafeteria scene. In the second recording context, participants again listened to single and dual speech narratives presented through experimental hearing aids. Here, they sat in a corridor outside the laboratory and walked freely outdoors, along the calm and busy city streets of Oldenburg, Germany. This recording context will be further referred to as Beyond-the-Lab (BTL). To investigate whether the outdoor condition had a detrimental effect on either EEG signal quality or attentional engagement (fatigue), a final recording took place in the laboratory speaker ring again (Lab 2), repeating the initial baseline context. As noted above, the cognitive strain may modulate the cortical tracking of continuously attended speech streams, and gross movements such as walking can be harmful to EEG signal quality (Jacobsen et al., 2020). With this in mind, we expected lower, but above chance, attention decoding results in Beyond-the-Lab free walking conditions, especially in dual-speaker listening scenarios. Due to the total length of the measurement and the complexity of the task, we participants are likely to experience fatigue at the end of the experiment. Therefore we also predicted that neural speech tracking will be less strong in listening conditions at the end (Lab 2) than the beginning (Lab 1) of the recording protocol, especially in challenging listening conditions.

## 2 Materials and methods

### 2.1 Participants

Twenty-eight participants took part in the study. Due to participant drop-out (*n* = 3), acute health problems (*n* = 1), neurological diseases (*n* = 2), compromised hearing (*n* = 1), and technical difficulties (*n* = 2) data from 20 participants were available for Lab 1 and Lab 2 (10 women) and data from 19 of the same participants for the Beyond-the-Lab block (nine women). The age of participants ranged between 19 and 31 years (median age: 24.5). Participants reported normal hearing and normal or corrected-to-normal vision. Participants signed an informed consent form and were compensated for their participation. The study was approved by the University of Oldenburg Ethics Committee (DRS.EK/2021/078).

### 2.2 Paradigm and measurement protocol

**Figure 1:**
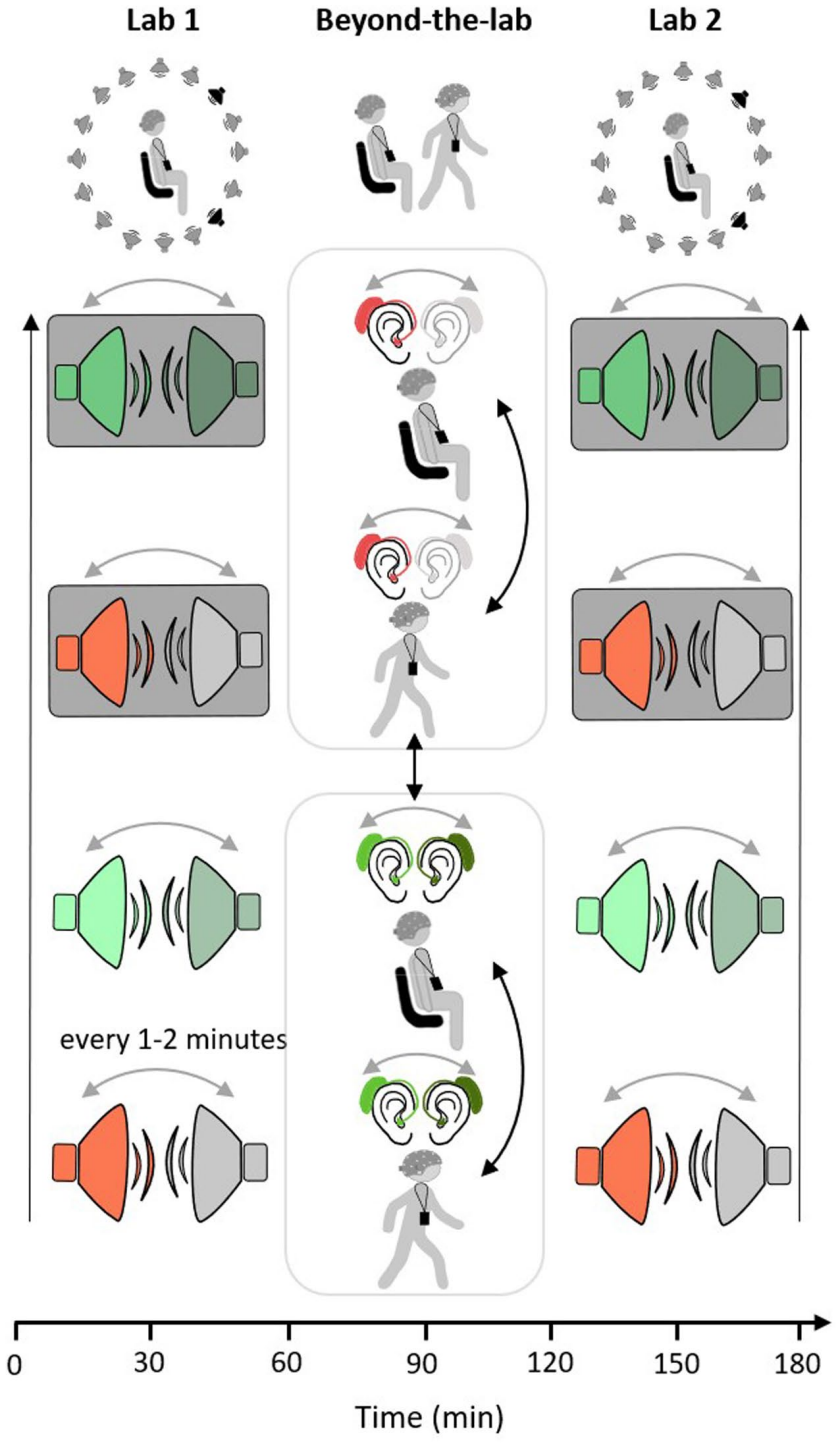
Measurement protocol: single-speaker conditions in red and dual-speaker conditions in green. Condition order in Lab 1 and Lab 2 measurement starts with single-speaker condition (red) and ends with a dual speaker with background cafeteria noise condition (green with grey background). Condition order does not change across participants or within measurement (black arrow, order of conditions from bottom to top). In the beyond the lab measurement the order of movement conditions (sit/walk) and listening conditions (single/dual) is pseudo-randomized across participants (black arrows). In all three measurement contexts, the side of the presentation of the attended speaker changes every 1 to 2 min (grey arrows).

Each participant was invited for two measurement appointments. One set-up consisted of a conventional 32-channel EEG cap, whereas another set-up included two arrays of the flex-printed cEEGrid electrodes attached behind both ears (cEEGrids, Bleichner and Debener, 2017; Debener et al., 2015). The second appointment took place approximately three weeks after the first. Both appointments took place on weekdays between 09:00 a.m. and 12:30 p.m. During both measurement appointments, the same measurement protocol was followed. Participants filled out an informed consent form and a questionnaire concerning their general wellbeing and their (neurological) health status. In the first measurement appointment, a pure tone audiogram measurement was conducted. This was followed by equipping the participant with the recording technology, including EEG and ECG hardware, the recording platform (see section 2.3.2.), and an Android smartphone. The measurement protocol consisted of three blocks (Lab 1, Beyond-the-Lab, Lab 2), which lasted approximately 60 min each. Lab 1 and Lab 2 took place in a laboratory of the Hörzentrum GmbH Oldenburg.^1^ After Lab 1, the Beyond-the-Lab (BTL) block was introduced, which took place outside of the laboratory. In all blocks, a single-speaker and a dual-speaker paradigm were employed (e.g., Mirkovic et al., 2016; O’Sullivan et al., 2015; Straetmans et al., 2021). Participants were instructed to attentively listen to one narrative read by a male or female speaker. Any other concurrently presented speech or noise should be ignored. In each of the three blocks, four consecutive narratives were presented, each lasting approximately nine min. After every narrative, participants were asked to answer four multiple-choice questions concerning the content of the presented story and to rate their level of fatigue and exhaustion on a scale from one to seven (1 = not tired at all, 7 = extremely tired; 1 = not exhausting at all, and 7 = extremely exhausting). In Lab 1 and Lab 2 blocks, participants were seated in the laboratory on a chair in the middle of a speaker ring consisting of 16 speakers. The level of acoustic complexity increased with each presented narrative as follows: The first story was read by one speaker without any further sources of distraction (single speaker). The second story was presented simultaneously with another, concurrently played narrative read by a speaker of the opposite sex of the attended narrative (dual speaker). Then, participants should again focus on only one speaker while a cafeteria scene was presented in the background (single speaker and background). In the fourth and last story, participants were presented with the attended narrative, a concurrently running narrative, and the cafeteria scene in the background (dual speaker and background). The order of presentation scenarios was kept constant across participants because one key objective was to induce listening fatigue by steadily increasing the level of acoustic complexity. In the Beyond-the-Lab block, participants were presented with one or two speaker stimuli while sitting on a chair in a relatively quiet hallway or walking a predefined route near the Hörzentrum Oldenburg. The route led along quiet (i.e., Küpkersweg) and busy streets (i.e., Ammerländer Heerstraße). The order of conditions was randomized across participants (see Figure 1).

### 2.3 Data recording

#### 2.3.1 Portable hearing lab (PHL)

**Figure 2:**
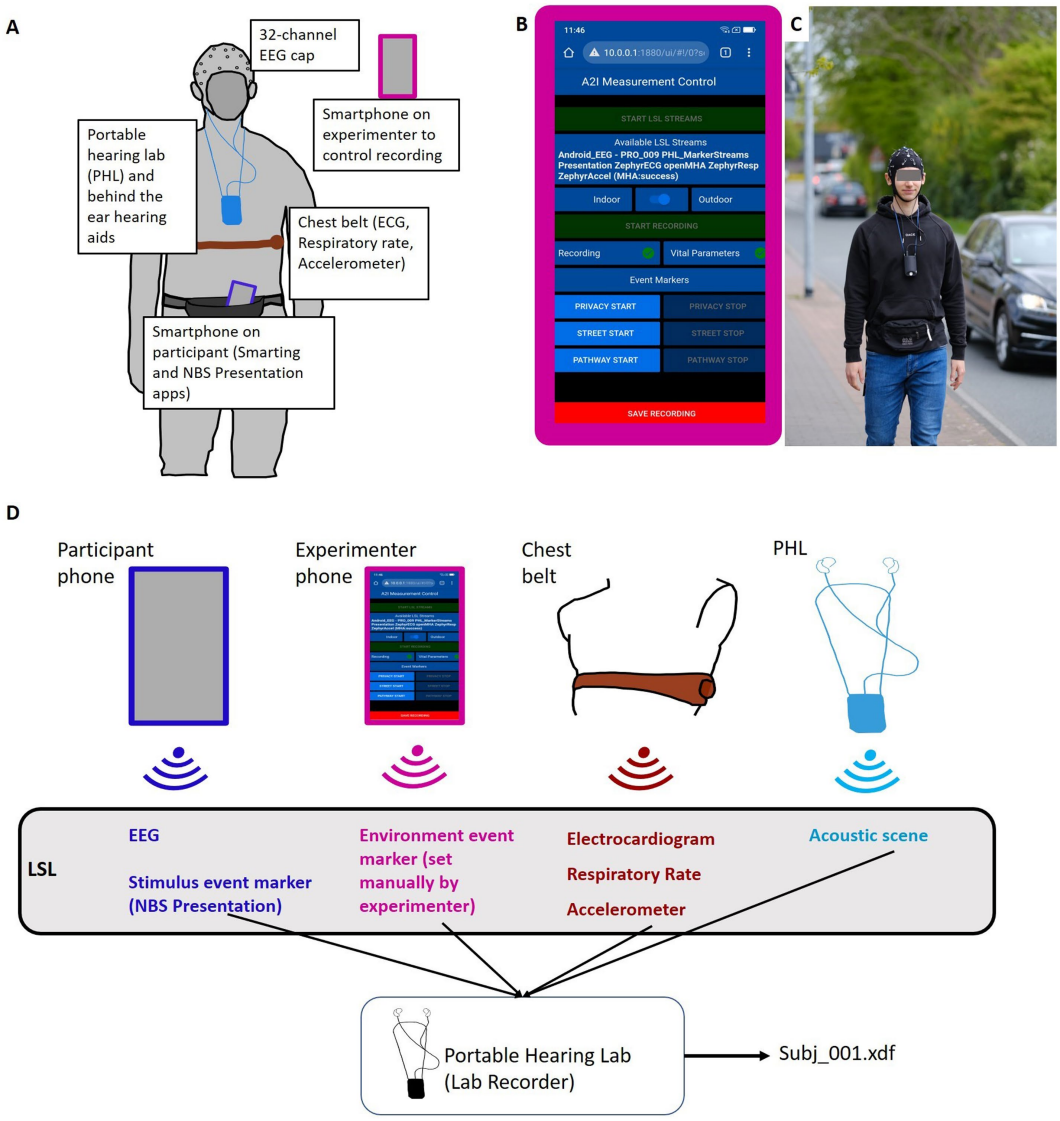
Study set up. (A) Pictogram of all included sensors and recording devices on the participant. (B) Node Red GUI on the experimenter’s phone to control the experiment and monitor LSL data stream availability. (C) Participant in BTL walking condition. (D) Pictogram of technical set-up. LSL streams of different devices are streamed via Wi-Fi and recorded on the PHL into an .xdf file.

The Portable Hearing Lab (PHL, https://batandcat.com/portable-hearing-laboratory-phl.html) is a small and lightweight hearing aid research platform (PHL, https://batandcat.com/portable-hearing-laboratory-phl.html) (see Figure 2). It consists of a BeagleBone Black wireless single-board computer, a battery, a multi-channel audio board, and two binaural behind-the-ear hearing aids as well as receivers located in the ear canal. The device has a measurement of 10 cm × 6.5 cm × 5 cm (hxwxd) and a weight of 166 g. It can be worn around the neck (see Figure 2). For the purpose of this study, the PHL allowed a mobile, time-synchronous recording of all data streams into one file. The device offers a Wi-Fi interface which allows communication with other computers or smartphones. The Wi-Fi interface enabled the wireless programming of a mobile phone app to control the device.

#### 2.3.2 Additional data streams

The study was conducted as part of a larger project^2^ that required the collection of vital signals. Information about the acoustic environment was also collected (see Figure 2). To capture electrocardiogram (ECG) signals, participants were equipped with a chest belt.^3^ The chest belt captured respiratory rate and accelerometer data. The results of the cardiovascular data recorded in this experiment will be presented elsewhere. Furthermore, the entire acoustic environment (i.e., presented speech signal and environmental noise) during the measurements was recorded via two microphones implemented in each behind-the-ear hearing aid. These audio recordings were transformed online into a lab streaming layer data stream for further synchronization.

#### 2.3.3 Data synchronization and recording

Stream synchronization and data recording were implemented with the Lab Streaming Layer software framework. The Lab Streaming Layer (LSL, Kothe et al., 2024) is a communication protocol designed to facilitate the real-time exchange of time-series data between various devices and software applications in a laboratory environment. LSL provides a standard to synchronize different data streams. LSL is particularly popular in neuroscientific research, where it is often used to stream data from different devices such as EEG, eye-tracking systems, motion capture systems, and other physiological sensors. Data streams can be received and time-synchronized with the LSL Lab Recorder. Lab Recorder is an application built on top of LSL that enables researchers to easily record, timestamp, and organize data streams from multiple sources during an experiment. With Lab Recorder, researchers can create synchronized recordings of diverse data modalities, capturing a comprehensive picture of experimental events. All collected data streams are saved into one .xdf file on the recording platform. Together, LSL and Lab Recorder provide a framework for collecting and managing multi-modal data in a standardized manner. In the current study, multiple data streams (EEG, vital parameters, event markers, and acoustic scenes) from different recording devices had to be recorded in a time-synchronous manner (Figure 2). For this purpose, data streams were converted to LSL streams and recorded on the PHL (see section 2.3.1.) via Lab Recorder (see Dasenbrock et al., 2022, for further details). During measurements, the experimenter controlled the PHL via a custom-made graphic user interface (GUI) implemented via NodeRed^4^ and running on a second smartphone (experimenter phone). This allowed us to set the date, time, and participant ID as well as start and save the recording. During data recording, the experimenter monitored all relevant data streams via the NodeRed GUI.

### 2.4 Stimuli

#### 2.4.1 Speech

Speech stimuli were edited with the Audacity software (v2.3.0, https://www.audacity.de/). The stimuli were pre-processed by removing the DC offset and normalizing the maximum amplitude to −1,0 dB. The normalization adjusts the peak level of the left and right channels in order to ensure comparability of the narratives. Additionally, silent moments within the narratives were truncated.

#### 2.4.2 Stimulus presentation

**Figure 3:**
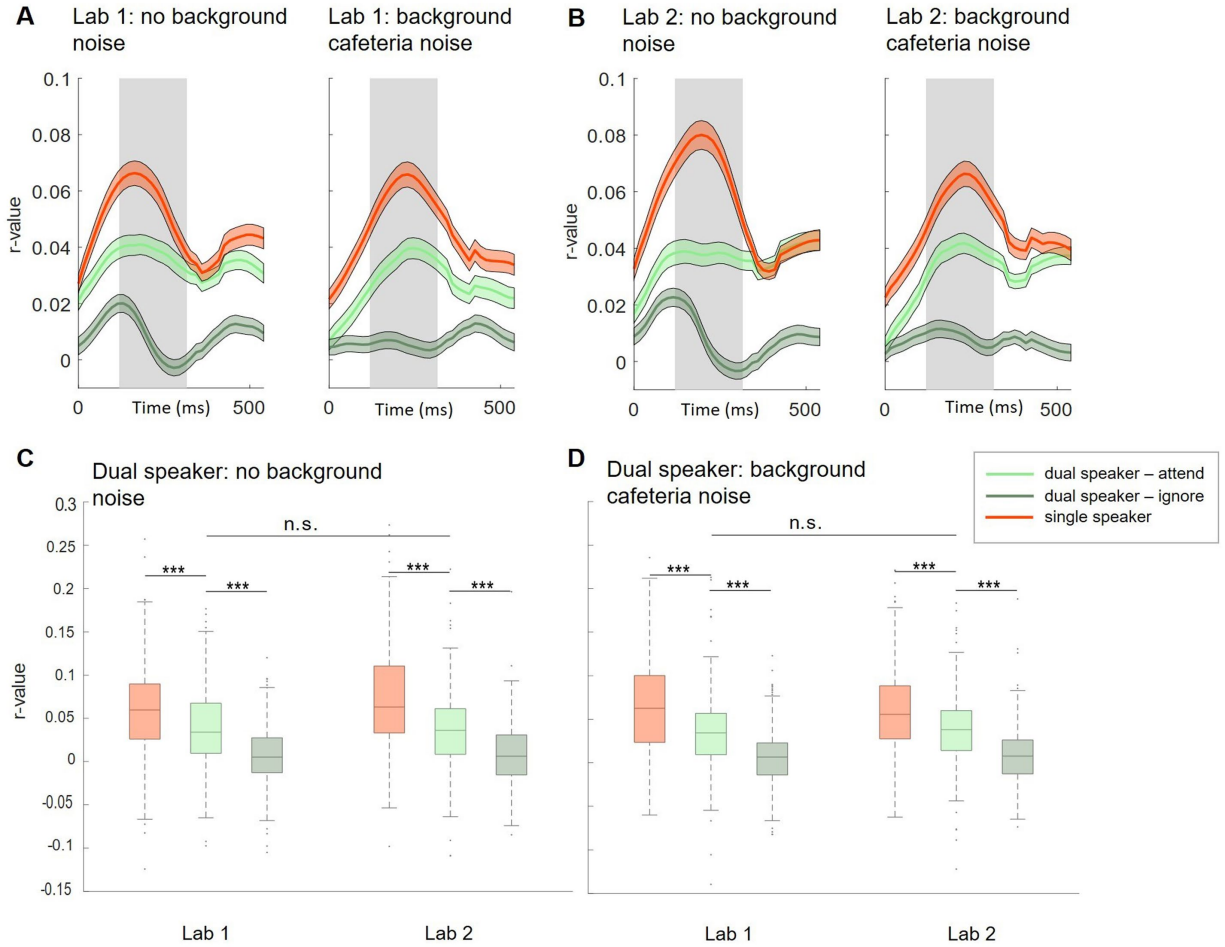
Subject-independent model, Lab 1 and Lab 2. (A,B) Morphology of neural tracking from 0 to 500 ms relative to the speech envelope. Displayed for single (red) and dual (attended: light green, ignored: dark green) speaker conditions in Lab 1 (A) and Lab 2 (B) measurements. Shaded colored areas show +/− 1 standard error. The shaded gray area represents the time window used for analysis. (C,D) Comparison of neural tracking within and between Lab 1 and Lab 2 single-and dual-speaker conditions (C: without background cafeteria noise; D: with background cafeteria noise) ***p < 0.001.

Participants were instructed to follow the narrative read by a male or a female speaker over the entire course of the experiment. During both appointments, the sex of the speaker the participant should should follow remained the same, however, the speaker and the content of the narrative differed. Lab 1 and Lab 2 measurements took place in a laboratory of the Hörzentrum Oldenburg equipped with a speaker ring (16 speakers). A toolbox for acoustic scene creation (TASCAR, Grimm et al., 2019) was used to present auditory stimuli. Event markers were generated with MATLAB and streamed via LSL. The to-be-attended narrative was presented via speakers located at an angle of −30° (left-side presentation) or 30° (right-side presentation) relative to the participant (see Figure 3). In the dual-speaker conditions, the to-be-ignored narrative was presented from the opposite side (left-side presentation: −30°, right-side presentation: 30°). In the single-speaker condition, no additional stimuli were presented from the opposite side. After varying time intervals of between 60 and 120 s, the to-be-attended and the to-be-ignored speaker sides of the presentation were switched. In the single-speaker condition, the to-be-attended speaker simply changed the side of the presentation after varying time intervals of between 60 and 120 s. Switches occurred at semantically meaningful moments within the stories.

In the Beyond-the-Lab block, auditory stimuli were presented via behind-the-ear (BTE) hearing aids consisting of two microphones placed above each ear. The audio was played via receivers inside the ear (see section 1.3.2.). The narratives were spatially separated by convolving the raw stimuli with head-related impulse responses (Kayser et al., 2009). The to-be-attended narrative was transformed to an angle of −30° (left-side presentation) or 30° (right-side presentation). In the dual-speaker condition, the to-be-ignored narrative was transformed to the opposite side. The presentation application from Neurobehavioral Systems Inc. (Albany, CA, United States) was used to play audio and stream event markers on an Android phone (Participant phone). At the beginning of Lab 1 and the BTL measurements participants were presented with a short audio snippet and asked to adjust the volume to a comfortable listening level. Participants either told the experimenter to adjust the volume level (Lab 1) or adjusted it themselves by pressing the volume buttons on the participant’s smartphone (BTL).

### 2.5 EEG acquisition

EEG was recorded with a wireless 32-channel direct current (DC) amplifier (SMARTING Pro, mBrainTrain, Belgrade, Serbia). The amplifier was attached to the back of the EEG cap (EasyCap GmbH, Herrsching, Germany). During the first appointment, the EEG of N = 10 participants was recorded with a 32 Ag/AgCI passive electrode montage (international 10–20 system, EasyCap, Berlin). In the second appointment, the same participants were equipped with two mobile electrode arrays called cEEGrids. The cEEGrid is a c-shaped array of ten flex-printed electrodes (AG/AgCI) (Debener et al., 2015). The other half of the participants (*N* = 10) were equipped with cEEGrids in the first appointment and with the 32-channel cap in the second appointment. The current study focuses on the cap data, cEEGrid results will be presented elsewhere. For cap measurements channel FCz served as a reference electrode. EEG was recorded with a sampling rate of 250 Hz. We prepared the electrode sites with alcohol and an abrasive electrolyte gel (Abralyt HiCl, EasyCap GmbH, Herrsching, Germany). Impedances were maintained below 10 kΩ. As data acquisition lasted for more than three h impedance was regularly checked and, if necessary, corrected during breaks. The EEG signal was transmitted wirelessly via Bluetooth to a smartphone (participant phone, Samsung S21) with a running Smarting application (v2, mBrainTrain, 2016, Fully Mobile EEG Devices). EEG data were converted into an LSL stream and sent into the PHL Wi-Fi network (see section 2.3.3.).

## 3 Analysis

### 3.1 Pre-processing

#### 3.1.1 EEG

Electroencephalography data were pre-processed and analyzed offline using MATLAB R2022b (MathWorks, Inc., Natick, United States) and EEGLAB v2022.0 (Delorme and Makeig, 2004). In the first step, data for each story were extracted from raw data recordings. For each participant and session, we had twelve segments consisting of 3 (Lab 1, BTL, Lab 2) × 4 (four stories presented in each block) data sets. Each recording lasted approximately nine min. Preprocessing and artifact reduction were carried out in two ways: in the first preprocessing, pipeline data were high-pass filtered at a cutoff frequency of 0.5 Hz (FIR, hamming, filter order 1652) and low-pass filtered at a cutoff frequency of 40 Hz (FIR, hamming, filter order 86). Then, the root mean square (RMS) of each channel was calculated and channels exceeding two standard deviations above the mean RMS were removed and interpolated. Additionally, bad data sections were identified based on high RMS values across multiple channels. Identified sections were removed from the data set (in two participants on average 5.1 s). In the second, more elaborate preprocessing pipeline, an additional Infomax independent component analysis (ICA) (Delorme and Makeig, 2004) was applied. ICA was conducted as follows. Data were first high-pass filtered at 1 Hz (FIR, hamming, filter order 828, using EEGLAB function *pop_eegfiltnew*), and noisy channels were removed. For each participant, data of all blocks were concatenated and cut into consecutive 1-s epochs. Epochs containing atypical artifacts were rejected based on probability [standard deviation steps (SD) = 3] and kurtosis criteria (SD = 3). ICA components reflecting stereotypical artifacts (i.e., eye blinks) were manually identified and removed by back-projecting the remaining components to the continuous raw data (on average 3.1 components removed). After ICA component-based data correction, previously removed channels were interpolated. In both pre-processing pipelines, data were subsequently low-pass filtered at a cutoff frequency of 15 Hz (FIR, hamming, filter order 222, *pop_eegfiltnew*), re-referenced to common average, downsampled to 64 Hz, and normalized by dividing them by their standard deviation.

#### 3.1.2 Speech envelopes

Speech envelopes were created using the function *mTRFenvelope* from the mTRF toolbox v2.3 (Crosse et al., 2016). This function extracts the envelope computing the root mean square (RMS) of the original audio signal (default window parameter = 1). To model human hearing, RMS intensity is logarithmically scaled to a power value of 0.3. Finally, the transformed signal is down-sampled to the new sampling rate of the EEG (64 Hz).

### 3.2 Auditory attention decoding

The human brain tracks low-frequency fluctuations of speech. As a consequence, features of these stimuli are represented in neural activity (Aiken and Picton, 2008; Ding and Simon, 2012a). Methods were developed to predict the neural response to known stimuli (encoding models) or reconstruct stimuli features from neural activity (decoding models). In the realm of cortical speech tracking, multivariate linear regression models are employed to relate features of speech stimuli to corresponding EEG (for a summary of auditory attention identification methods see Alickovic et al., 2019). In the current study, the multivariate Temporal Response Function (mTRF) Toolbox (v2.3) was used to evaluate the association between neural signals and speech (Crosse et al., 2016). A multivariate linear regression decoding model was trained on the attended envelope using time lags between 0 and 500 ms with a 45-ms moving window and 30-ms overlap. Trained decoders were applied to reconstruct the attended and ignored speech envelopes with no knowledge of the original stream itself. The magnitude of neural tracking across different listening conditions was evaluated based on the resulting correlation values between reconstructed and original speech envelopes (Pearson correlation). Decoding performance was quantified by the percentage of trials in which the reconstructed stimulus correlated more strongly with the original attended than with the original ignored stimulus (decoding accuracy). The chance level of decoding accuracies was based on a binomial significance threshold at an alpha confidence limit of 0.05. As in the current study the amount of data per subject and condition was relatively low (nine min per subject and condition; cf. Crosse et al., 2021), subject-dependent and subject-independent models were employed and evaluated. Subject-dependent models typically result in better model performance, yet model generalizability suffers if the sample size per subject and condition is small. Alternatively, subject-independent models can be designed to improve model generalization, yet model performance is typically lower (Crosse et al., 2021).

#### 3.2.1 Subject-independent model

For each subject and condition, EEG data and envelopes of attended and ignored speech were partitioned into non-overlapping 60-s data segments (*mTRFpartition*). For every condition, EEG data as well as speech envelopes of all subjects were concatenated into three vectors, respectively. A leave-one-out cross-validation was performed to determine the optimal regularization parameter (lambda) from a given set of seven values ranging from 10^−6 to 10^+6 (*mTRFcrossval*). For this purpose, data were partitioned into 9*20 (in BTL condition 9*19) folds. Each fold contained 1 min of data. One of the folds was held out as a test set, while the rest served as training set. The lambda corresponding to the maximum correlation value was used to train the model on the attended speech envelope and the corresponding EEG (*mTRFtrain*). Finally, the model of the attended speech envelopes was tested using the held-out test set of attended and ignored speech envelopes, respectively (*mTRFpredict*). This resulted in correlation values between the reconstructed and the original attended and ignored speech envelope for 34 time lags between 0 and 500 ms for every trial. These model statistics were used to assess differences in neural tracking across different listening conditions.

#### 3.2.2 Subject-dependent model

Electroencephalography data and envelopes of attended and ignored speech were again partitioned into non-overlapping 60-s data segments. This resulted in a total of nine data segments per condition. An iterative 9-fold cross-validation procedure was performed to determine the smoothing parameter lambda. The remaining data segment was held out as a test set to validate the model. For cross-validation, the same set of lambda values as used in the subject-independent model was applied. Then, a decoder was trained on the training set and tested on the left-out test set. Model testing was performed on the attended and the ignored speech envelopes, respectively. Subject-dependent model results were compared for different neural tracking conditions and used to investigate subject-specific differences in neural tracking estimates.

### 3.3 Robustness of neural tracking

We also evaluated the robustness of neural tracking across conditions, by analyzing correlations of neural tracking between different challenging acoustic scenarios. For this purpose, participants were ranked according to neural tracking magnitude within each condition. Next rankings between conditions were correlated. High correlations between two conditions indicate robust neural tracking of the attended speaker within subjects across different listening contexts, suggesting a low influence or even absence of detrimental effects of listening condition or movement.

### 3.4 Neural tracking in quiet and busy streets

Participants walked along a mostly quiet street (Küpkersweg, Oldenburg) leading toward the busier main street (Ammerländer Heerstraße, Oldenburg). They walked along the main street for approximately two min before turning onto a less busy street again. During data collection, the experimenter sent an event marker whenever the participant entered and left the main street. The mix of environmental noise and the simultaneously presented speech signal was recorded via microphones located behind the ears of the participants. Based on the manual markers we extracted two two-minute segments of signal + noise audio recording. The first segment was extracted from the beginning of the BTL measurement where participants walked along the quiet. This segment was labeled ‘quiet street’. The second segment started when participants turned onto the main street was labeled ‘busy street’. Then, neural tracking results of the subject-dependent decoding model during these segments were extracted. This way it was possible to explore whether neural tracking systematically differs in quiet compared to busy streets.

## 4 Results

### 4.1 Questionnaire data

After each story, participants answered questions concerning the content of the presented audio. Additionally, participants indicated their level of tiredness and exhaustion on a scale from one to eight (1 = not at all, 7 = extremely tired/exhausted). In the Lab 1 measure, average scores of correct answers to content questions ranged from 3.15 to 3.55 (Table 1). The lowest scores were obtained for the dual-speaker condition with cafeteria background noise. Average tiredness ranged from 3.15 to 3.85 (3 = a little tired, 4 = medium tired, Table 1) and average exhaustion ranged from 1.8 to 4.35 (1 = effortless, Table 1). High tiredness and exhaustion were reported for the dual-speaker condition with background noise. In the Lab 2 measure, average scores on content questions ranged from 2.7 to 3.1. The lowest score was observed for the dual-speaker condition without background noise. Tiredness ranged on average between 3.4 and 3.9 and average exhaustion between 1.7 and 4.15. The highest tiredness scores were observed for the single-and dual-speaker conditions with background noise. Average exhaustion was highest for the dual-speaker condition with background noise. In the BTL block average scores on content questions were found between 1.95 and 3.47 with the lowest scores for the dual-speaker walking condition. Average tiredness ranged from 2.21 to 3.05 (2 = very little tired, 3 = a little tired). The highest tiredness was observed for the dual-speaker sitting condition. A different picture was observed for average exhaustion. Here, scores ranged between 1.84 and 6.11 (6 = very exhausting), and the highest score was obtained in the dual-speaker walking condition.

**Table 1:** Group average results of questionnaires (content of narrative, tiredness, and exhaustion) for different listening conditions in Lab 1, Lab 2, and BTL measurements, respectively.

Block	Listening condition	Content (max 4)	Tiredness (1 = not at all; 8 = extreme)	Exhaustion (1 = not at all; 8 = extreme)
Lab 1	Single	3.35	3.15	1.8
	Dual	3.45	3.05	3.6
	Single + background	3.55	3.25	3.15
	Dual + background	3.15	3.85	4.35
Lab 2	Single	3.65	3.4	1.7
	Dual	2.7	3.7	3.1
	Single + background	2.95	3.9	3.35
	Dual + background	3.1	3.9	4.15
BTL	Sit single	3.47	2.58	1.84
	Sit dual	2.89	3.05	3.74
	Walk single	2.47	2.21	4.21
	Walk dual	1.95	2.26	6.11

### 4.2 Auditory attention decoding

#### 4.2.1 Subject-independent model

**Figure 4:**
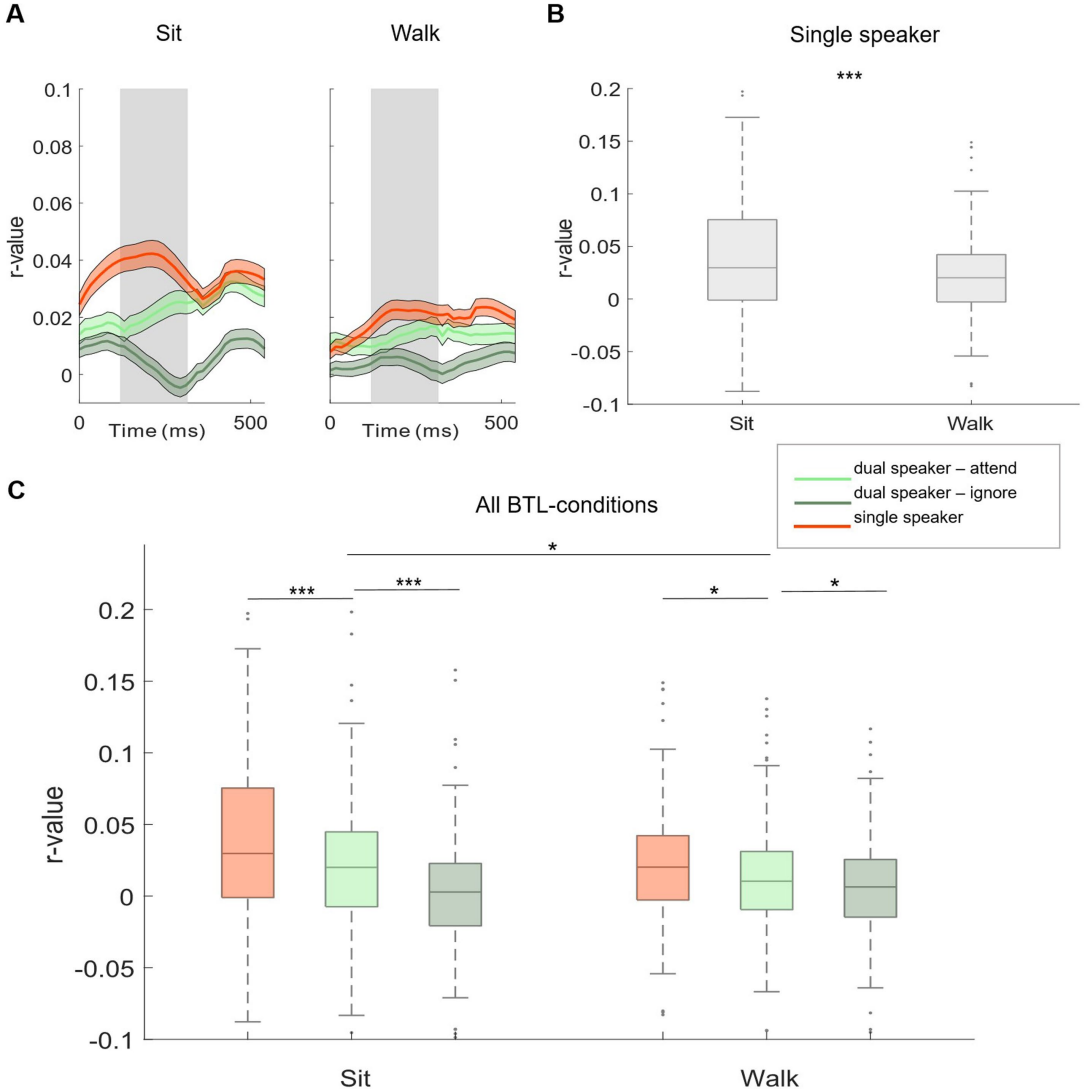
Subject-independent model, BTL. (A) Morphology of neural tracking from 0 to 500 ms relative to the speech envelope. Displayed for single (red) and dual speaker (attended: light green, ignored: dark green) conditions in BTL movement conditions (sit and walk). The shaded gray area represents the time window used for analysis. (B) Comparison of single-speaker conditions between BTL movement conditions. (C) Comparison of single-and dual-speaker listening conditions within and between BTL movement conditions. Lines between boxplots show single-subject statistics. Shaded colored areas show +/− 1 standard error. * *p* < 0.05. ** *p* < 0.01. *** *p* < 0.001.

Based on neural tracking morphology, time lags between 120 and 270 ms after stimulus onset were used for the analysis (Figures 3A,B, shaded gray area). Within Lab 1 and Lab 2 measurements, we observed significantly higher neural tracking estimates for single-speaker listening conditions than listening conditions in which two speakers were presented simultaneously. This was seen for the easier listening conditions and for the more complex scenarios including cafeteria background noise (Lab 1: Figure 3C, no cafeteria noise: Wilcoxon signed-rank test *Z* = 4.73, *p* < 0.001; Figure 3D: with cafeteria noise: Wilcoxon signed-rank test *Z* = 5.55, *p* < 0.001; Lab 2: Figure 3C, no cafeteria noise: Wilcoxon signed-rank test *Z* = 7.52, *p* < 0.001, Figure 3D: with cafeteria noise: Wilcoxon signed-rank test *Z* = 5.37, *p* < 0.001). A similar pattern of results was observed for the difference in neural tracking between the attended and the ignored speaker in the dual-speaker conditions. In both Lab 1 and Lab 2 measures, significantly higher neural tracking of the attended speaker than the ignored speaker was evident. Again, this effect was found for the easier and the more complex listening conditions (Lab 1: Figure 3C, no cafeteria noise: Wilcoxon signed-rank test *Z* = 6.83, *p* < 0.001, Figure 3D: with cafeteria noise: Wilcoxon signed-rank test *Z* = 6.86, *p* < 0.001; Lab 2: Figure 3C, no cafeteria noise: Wilcoxon signed-rank test *Z* = 6.87, *p* < 0.001, Figure 3D, with cafeteria noise: Wilcoxon signed-rank test *Z* = 7.51, *p* < 0.001). The difference in neural tracking between single-speaker with and without cafeteria background noise was not significant in Lab 1 or Lab 2 measures. For dual-speaker conditions, we additionally calculated average decoding accuracies representing the percentage of correctly reconstructed trials. In the laboratory recordings without background noise, 76.1% of trials were reconstructed correctly in Lab 1. In Lab 2, decoding accuracy was at 78.89%. In conditions with background cafeteria noise, the maximum decoding accuracies were at 75 and 78.9%, respectively (statistical chance level: 54.4%). Neural tracking in the BTL condition was analyzed in the same time lags as for Lab 1 and Lab 2 (between 120 and 270 ms after stimulus onset, see Figure 4A). When looking into results for the BTL measure, we found significantly higher neural tracking estimates for single-speaker conditions than dual-speaker conditions. This pattern was evident in sitting and walking conditions (Figure 4C, sit: Wilcoxon signed-rank test *Z* = 3.75, *p* < 0.001; walk: Wilcoxon signed-rank test *Z* = 2.41, *p* = 0.016). In both movement conditions, neural tracking of the attended speaker was systematically higher than tracking of the ignored speaker (Figure 4C, sit: Wilcoxon signed-rank test *Z* = 4.82, *p* < 0.001; walk: Wilcoxon signed-rank test *Z* = 2.45, *p* = 0.014). As for single-speaker conditions, neural tracking magnitude was higher in the sitting than the walking condition (Figure 4B: Wilcoxon signed-rank test Z = 3.76, *p* < 0.001). A similar pattern occurred in dual-speaker conditions. Here, neural tracking of the attended speaker in the sitting condition was higher than in the walking condition (Figure 4C: Wilcoxon signed-rank test *Z* = 2.26, *p* = 0.024). Average decoding accuracies reached up to 71.4% in the sitting condition and 63.2% in the walking condition (chance level: 54.4%).

#### 4.2.2 Subject-independent model with ICA

When data were cleaned with ICA, we found significantly higher levels of neural tracking in single-speaker conditions of Lab 1 than Lab 2 single-speaker conditions (without background noise: Wilcoxon signed-rank test *Z* = −2.14, *p* = 0.03; with background noise: *Z* = −2.28, *p* = 0.02). A similar pattern was observed for dual-speaker conditions. Here, results confirmed significantly higher neural tracking estimates for the attended speaker in the dual-speaker condition in Lab 1 than Lab 2 (see Supplementary Figures S4, S5). This difference was larger in dual-speaker conditions with background noise (with background noise: Wilcoxon signed-rank test *Z* = −3.88, *p* = 0.0001; without background noise: *Z* = −1.98, *p* = 0.05). As for the BTL measure, when data were cleaned with ICA the difference in neural tracking between sitting and walking conditions was more pronounced. This was observed for single-and dual-speaker conditions (single speaker: Wilcoxon signed-rank test *Z* = 4.57, *p* > 0.0001, dual speaker: *Z* = 5.13, *p* > 0.0001). Within the walking condition, results indicate a stronger difference in neural speech tracking between the attended speaker in single-and dual-speaker conditions when ICA cleaned data are used (Wilcoxon signed-rank test *Z* = 3.38, *p* > 0.001). As results with the less elaborate pre-processing pipeline are still satisfactory and comparable to results obtained with ICA-cleaned data, further analyses were performed on data cleaned via the less elaborate and quicker preprocessing pipeline.

#### 4.2.3 Subject-dependent model

Neural tracking results were generally higher for the subject-dependent model (see Supplementary Figures S1, S2). For Lab 1 and Lab 2, results between subject-dependent and subject-independent models were comparable. As for the BTL measure, we found a larger difference between attended and ignored speakers in the walking condition for the subject-dependent model (Wilcoxon signed-rank test *Z* = 3.46, *p* < 0.01).

### 4.3 Robustness of neural tracking

**Figure 5:**
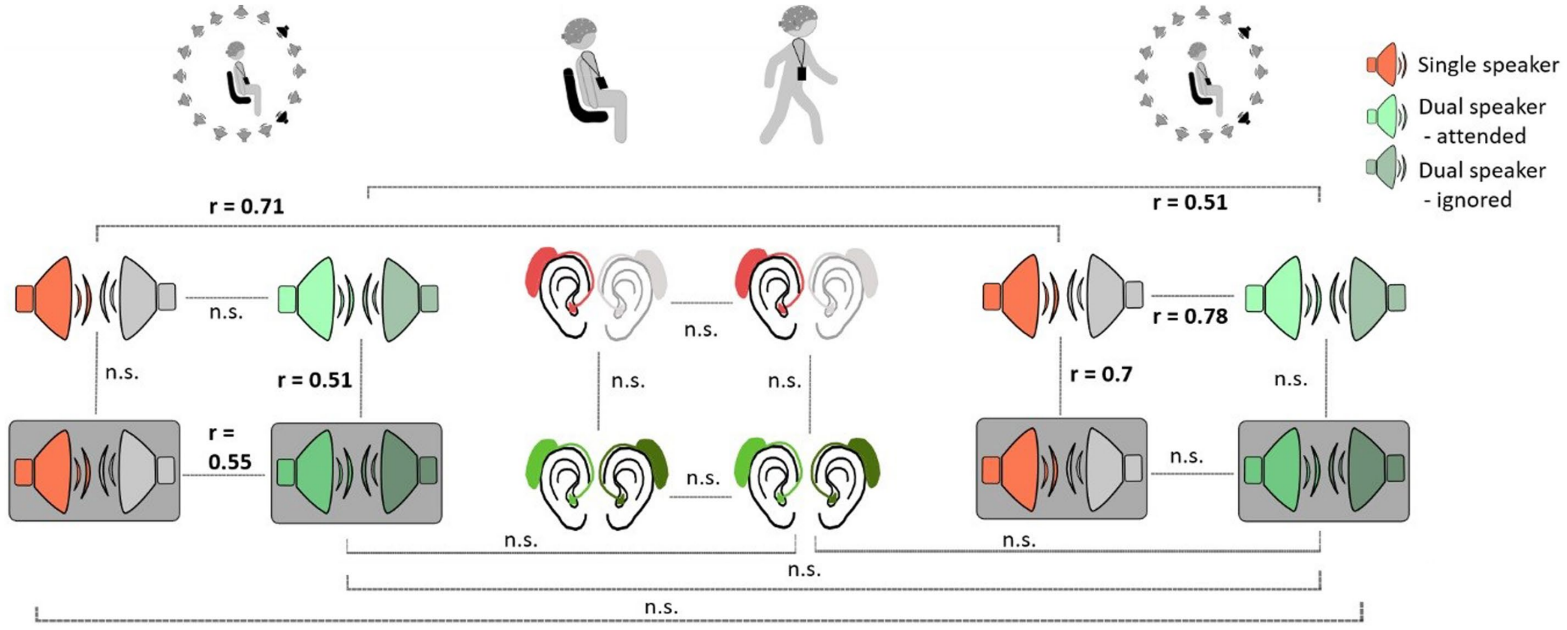
Within-subject robustness of neural tracking between different listening conditions and measurement context. Grey boxes represent conditions with background noise.

The following within-subject analyses were performed with neural tracking results of the subject-dependent model. When comparing the robustness of neural tracking for participants across listening conditions and contexts, we obtained mixed results. We observed significant rank correlations between different listening conditions within and between Lab 1 and Lab 2 measurements (Figure 5). In Lab 1, neural tracking between different listening conditions seemed to be more robust for more complex conditions. Here, rank correlations between the single-speaker condition with background noise and the dual-speaker condition with background noise were significant (*r* = 0.55, *p* = 0.02) as well as between the dual-speaker condition without background noise and the dual-speaker condition with background noise (*r* = 0.51, *p* = 0.03). Interestingly, we also found significant rank correlations between less complex listening conditions in Lab 2 measurements. Correlations between the single-speaker condition without and the single-speaker condition with background noise were significant (*r* = 0.71, *p* = 0.001). Moreover, a significant association between ranks in the single-speaker condition without background and the dual-speaker condition without background was observed. To explore neural tracking robustness over time, ranked neural tracking results in conditions of Lab 1 measurements were compared to those in the Lab 2 measurement. Here, rank correlations between Lab 1 and Lab 2 in single-and dual-speaker conditions without background noise were significant (single speaker: *r* = 0.71, *p* = 0.001; dual speaker *r* = 0.51, *p* = 0.03). There was no significant rank correlation within the BTL measure or between Lab 1 or Lab 2 and the BTL measure.

### 4.4 Neural tracking in quiet and busy streets

We also explored whether neural tracking was different for data recorded in busy compared to calm street segments. On a group average level, we did not find a significant difference in neural tracking between busy and calm street conditions (calm vs. busy street: single-speaker condition Wilcoxon signed-rank test *Z* = 0.04, *p* = 0.97; dual-speaker condition *Z* = 0.89, *p* = 0.38). Then, we also looked into rank correlations between busy and calm streets in order to test whether neural tracking remains robust across busy and calm street sections in single-and dual-speaker conditions for individual participants. Here, we also did not find significant associations (calm vs. busy street: single-speaker condition *r* = 0.19, *p* = 0.43; dual-speaker condition *r* = 0.14, *p* = 0.57).

## 5 Discussion

We used a commonly employed single-and dual-speaker paradigm to study auditory attention in real-life contexts. Single-and dual-speaker scenarios were presented in quiet and noisy situations inside and outside of the laboratory, while participants were taking a walk along quiet and busy streets. This study aimed to test whether EEG-based auditory attention decoding is feasible under conditions with multiple sources of distraction and movement.

In order to ensure that participants followed the to-be-attended speaker they were asked to answer multiple-choice questions about the content of the most recently presented story. Participants were also instructed to rate their subjective level of tiredness and exhaustion. The results of the content questions show that in the laboratory blocks participants were on average able to answer the majority of questions correctly, similar to previous laboratory studies employing this paradigm (Jaeger et al., 2020; Mirkovic et al., 2015). In the BTL block, the results were mixed. While on average three out of four questions were answered correctly in the single-speaker sitting condition, lower performance was observed in the single-speaker walking condition. The walking condition with two simultaneous speakers resulted in the lowest number of correctly answered content questions on average, indicating that participants had listening difficulties or were highly distracted by real-life events occurring in the walking conditions. We suggest that uncontrolled ambient noise may have temporarily masked the speech stimuli. Reported tiredness ranged, on average, between “a little tired” and “medium tired” for all conditions within both laboratory blocks. Despite the length of the recording, participants did not feel excessively tired at the end of the measurement. Tiredness was rated lowest in the BTL walking conditions, most likely due to walking in fresh air. In terms of subjective exhaustion, the results show that single-speaker conditions in the laboratory and BTL blocks were rated on average less exhausting. Participants rated the dual-speaker condition with simultaneous background noise as the most exhausting laboratory condition. In the BTL measurement, the dual-speaker walking condition was perceived as the most exhausting even though it was rated as at least tiring. These results clearly indicate that participants were challenged by the task context, as intended. The pattern of the results suggests multiple influences of complex listening contexts on subjective wellbeing. However, despite the high ratings of exhaustion reported, we found clear evidence of attention-driven neural speech tracking even in the most complex listening conditions.

To investigate neural speech tracking, subject-independent and subject-dependent multilinear decoding models have been developed (Alickovic et al., 2019). Typically, subject-dependent models perform better as they can account for intersubject variability in neural responses (Crosse et al., 2021). However, due to the small amount of data per subject and condition, the generalizability of model results is modest (Crosse et al., 2021). Therefore, a subject-independent model was trained using data from all subjects within each listening condition.

In the Lab 1 and Lab 2 measurements, both models showed stronger neural tracking for to-be-attended speakers in single-speaker than dual-speaker conditions. This was found for conditions with and without background noise. Neural tracking estimates of both models were on average significantly higher for the to-be-attended than for the to-be-ignored speaker in dual-speaker conditions with and without background cafeteria noise. No significant differences were observed between Lab 1 and Lab 2 conditions. Decoding accuracies, as measured by the percentage of trials in which the reconstructed stimulus correlated more strongly with the original attended stream than with the original ignored stream, were above the chance level for both models in all dual-speaker conditions. Taken together, these results suggest that processing one instead of two speakers is reflected in a decrease in neural tracking of the attended speaker. Even though the second speaker was irrelevant to the task. The distraction induced by the second speaker as well as the additional load on the auditory system to identify and segregate specific features of the to-be-attended speaker may have driven this effect (O’Sullivan et al., 2019; Shinn-Cunningham and Best, 2008). Interestingly, decoding accuracy was generally higher in the two-speaker condition with cafeteria background noise than in the two-speaker condition without cafeteria noise. The mixture of the ignored speaker and the background noise as to-be-ignored distractors may have been spectrally less similar to the to-be-attended speaker than in the dual-speaker condition with just one competing speaker. The to-be-attended speaker may have been easier to separate from the background noise as a perceptual object and therefore easier to attend to (Shinn-Cunningham and Best, 2008).

In the next step, we investigated the influence of more or less extensive preprocessing on the performance of the subject-independent model. We compared model results based on minimally pre-processed data with model results based on data that had undergone an ICA. While the results remained similar within Lab 1 and Lab 2 measurements, we found significant differences between Lab 1 and Lab 2 measurements. In both single-and dual-speaker conditions, we find higher neural tracking estimates in the Lab 2 conditions. One explanation could be that as the recordings lasted on average approximately 3 h, ICA might reduce some artifacts related to the long recording duration. Taken together, our results support previous findings in that it is possible to correctly identify an attended speaker within a dual-speaker listening condition based on EEG (Mirkovic et al., 2015; O’Sullivan et al., 2015). Furthermore, we find that even in more complex scenarios with a background scene, significantly higher neural tracking of the attended speaker can still be shown (Fuglsang et al., 2017). Decoding accuracies even suggest a facilitating effect on attention in moderately complex and engaging listening contexts (Herrmann and Johnsrude, 2020). On average neural tracking seemed to improve from Lab 1 to Lab 2 measurements. Participants may have been more engaged and motivated to attentively follow the story attentively after being presented with the same consecutive narrative throughout the experiment, or they may have simply benefitted from taking a walk between Lab1 and Lab2.

In general, the results of the subject-independent and the subject-dependent models are more mixed and less comparable in the BTL measurement than the laboratory measures (Lab 1 and Lab 2). This may be explained by the fact that differences between the subject-dependent and subject-independent models are typically driven by inter-subject variability and that mobile EEG recordings outside the laboratory are naturally subject to more inter-subject variability due to subject-specific motion artifacts and uncontrollable environmental distractions. Similar to the results of Lab 1 and Lab 2, in the BTL-sitting condition the subject-independent model showed significantly higher neural tracking of the single-speaker condition than the neural tracking of the to-be-attended speaker in the dual-speaker condition. We did not find this pattern in the results of the subject-dependent model. Interestingly, although the neural tracking of the attended speaker was significantly higher than that of the ignored speaker in the walking condition, the difference was more pronounced in the subject-dependent model.

Confirming and extending our previous study (Straetmans et al., 2022), neural tracking of the single speaker and attended speaker tracking in dual-speaker conditions was higher in the sitting than in the walking conditions. This was found for both the subject-independent and the subject-dependent model. While decoding accuracies were above chance in both conditions, several factors may contribute to lower neural tracking in mobile recording conditions. First, EEG data in walking conditions may still be contaminated with residual motion artifacts (Jacobsen et al., 2020). Second, natural walking along city streets may have distracted participants from the auditory attention task, resulting in lower neural tracking estimates (Straetmans et al., 2021). Finally, the additional cognitive processes associated with walking may reduce the resources available for a cognitive task such as auditory attention (De Vos et al., 2014; Reiser et al., 2020). Along the same lines, Ladouce et al. (2019) suggest that the processing of movement-related visual and inertial stimuli may play a role. Given limited attentional resources, performing cognitive and motor tasks simultaneously may lead to cognitive motor interference (Al-Yahya et al., 2011; Leone et al., 2017). In the current study, a complex and realistic environment required the walking participant to monitor and process additional, potentially relevant information, such as bicycles on the path or cars on the road. All of these additional tasks may have led to the reduction in neural tracking during walking that we see in our data. Although the results of the current study are comparable to the results of our previous mobile attention decoding study, in the previous study decoding accuracies for sitting and walking conditions were generally higher and differences between sitting and walking conditions were not as pronounced as in the current study. One reason for this may be that the previous study took place in a large empty cafeteria hall with a level floor and no other sources of uncontrolled visual or auditory distraction. An easier movement condition may result in less cognitive-motor interference (Reiser et al., 2020). In addition, terrain complexity has been shown to influence gait control demands, which may also help to understand the differences between our previous and current studies (Jacobsen et al., 2022). However, the most likely factor that may have played a role is the mode of audio presentation. In the first study, audio was presented through noise-shielding in-ear headphones, whereas in the current study, small receivers placed in the ear canal with virtually no noise-shielding capacity were used. Given the goal of robust auditory attention tracking in real-life environments, future studies should be designed to disentangle the contributions from motor, perceptual, and cognitive domains.

We also compared the results obtained for minimally pre-processed and more rigorously pre-processed EEG data. While several factors could be at play, we conclude that ICA processing does not appear to have beneficial effects on neural tracking estimates in the walking condition. Similar to previous study (Jaeger et al., 2020; Straetmans et al., 2021), it appears that simple processing pipelines work well for neural tracking, even in mobile scenarios.

### 5.1 Robustness of neural tracking

We expected that if participants were able to listen attentively to two consecutive stories without disengaging in between, the neural tracking estimates between the two stories would be similar within this participant. However, if one story captured attention better than the second, participants may have been more prone to mind-wandering or boredom. As a result, neural tracking between these two stories may be more variable. In this analysis, we compared the rank correlations of different participants across different listening conditions in order to explore whether attention is stable across listening demands for individual participants. We found that listeners showed stable neural tracking performance across the more complex listening conditions during Lab 1 measures (i.e., dual speaker–dual speaker with background; single speaker with background–dual speaker with background). Interestingly, a similar pattern was observed in the Lab 2 measure but for the less demanding listening conditions (i.e., single speaker–dual speaker, single speaker–single speaker with background). We interpret this result as being due to resource exhaustion over the course of the long measurement. In Lab 1, the less complex listening conditions may have not have demanded sufficient resources for participants to focus narrowly on the task at hand. Due to this under-stimulation, attention may have wandered (Herrmann and Johnsrude, 2020) and neural tracking of the attended speaker was variable across the less complex listening conditions. This may have shifted in Lab 2 where attention resources are lower and may only allow for targeted processing of the less complex listening conditions. Here, a certain resource limit may have been reached, and sustained attention to the attended speaker in more complex listening conditions was more difficult and therefore more variable across participants.

### 5.2 Neural tracking in quiet and busy streets

We also examined neural tracking estimates during busy and quiet background street noise conditions. Contrary to our expectation that neural tracking should be lower when participants walked along busy streets, we found no clear differences. Note that background noise was not manipulated but given in these recording conditions. Due to the instantaneous activity on the quiet and busy street segments, they were in the end highly similar for some participants. In addition, the duration of the recording may have been too short to detect any effects. On a positive note, neural tracking was still possible in uncontrolled, qualitatively more or less complex auditory environments.

### 5.3 Conclusion and outlook

This study demonstrates that EEG-based auditory attention decoding can be successfully performed on data recorded in mobile, uncontrolled real-life situations. Interestingly, this was possible without any computationally intensive pre-processing of the EEG data. While the acquisition of good quality EEG signals outside the laboratory remains a challenge, our results complement other recent study reporting the successful acquisition of meaningful brain electrical signals during full body movement (e.g., Klapprott and Debener, 2024; Papin et al., 2024).

Specifically, our results suggest that neural measures of auditory attention can be identified in several different natural listening situations. Compared to previous study on auditory attention in mobile situations (e.g., De Vos et al., 2014; Debener et al., 2012; Ladouce et al., 2019), our study extracts neural signatures of attention to continuous natural stimuli, rather than discrete, artificial stimuli. EEG monitoring of attention to speech in noisy environments could have several applications, such as in educational settings, in various workplaces, or through the advancement of assistive technologies such as hearing aids. However, to achieve these goals, the technological readiness of mobile, motion-tolerant EEG systems needs to be improved (cf. Bleichner and Debener, 2017). It remains to be shown whether similar results can be achieved with unobtrusive ear EEG systems (Bleichner et al., 2017; Holtze et al., 2022), which could be worn for longer periods of time. Furthermore, online, near real-time auditory attention decoding remains a challenge (Geirnaert et al., 2022; Jaeger et al., 2020), as is the case with self-application of EEG systems (da Silva Souto et al., 2022). Finally, alternative, computationally sparse decoding models that do not rely on *a priori* known, clean streams or spatial locations (Geirnaert et al., 2021a) of auditory objects are needed to move mobile EEG-based attention for speech decoding from basic research to real-world application.

## Data Availability

The raw data supporting the conclusions of this article will be made available by the authors, without undue reservation.

## Footnotes

^1^ https://www.hz-ol.de/de

^2^ https://a2i-med.net/

^3^ https://www.zephyranywhere.com/system/overview

^4^ https://nodered.org/
