# Supplementary material for: Neural speech tracking and auditory attention decoding in everyday life
Source: Front Hum Neurosci. 2024 Nov 13;18:1483024. doi: 10.3389/fnhum.2024.1483024 (PMC11599177; doi:10.3389/fnhum.2024.1483024)
Supplement: Supplementary file 1 [file Data_Sheet_1.pdf]

Supplementary material

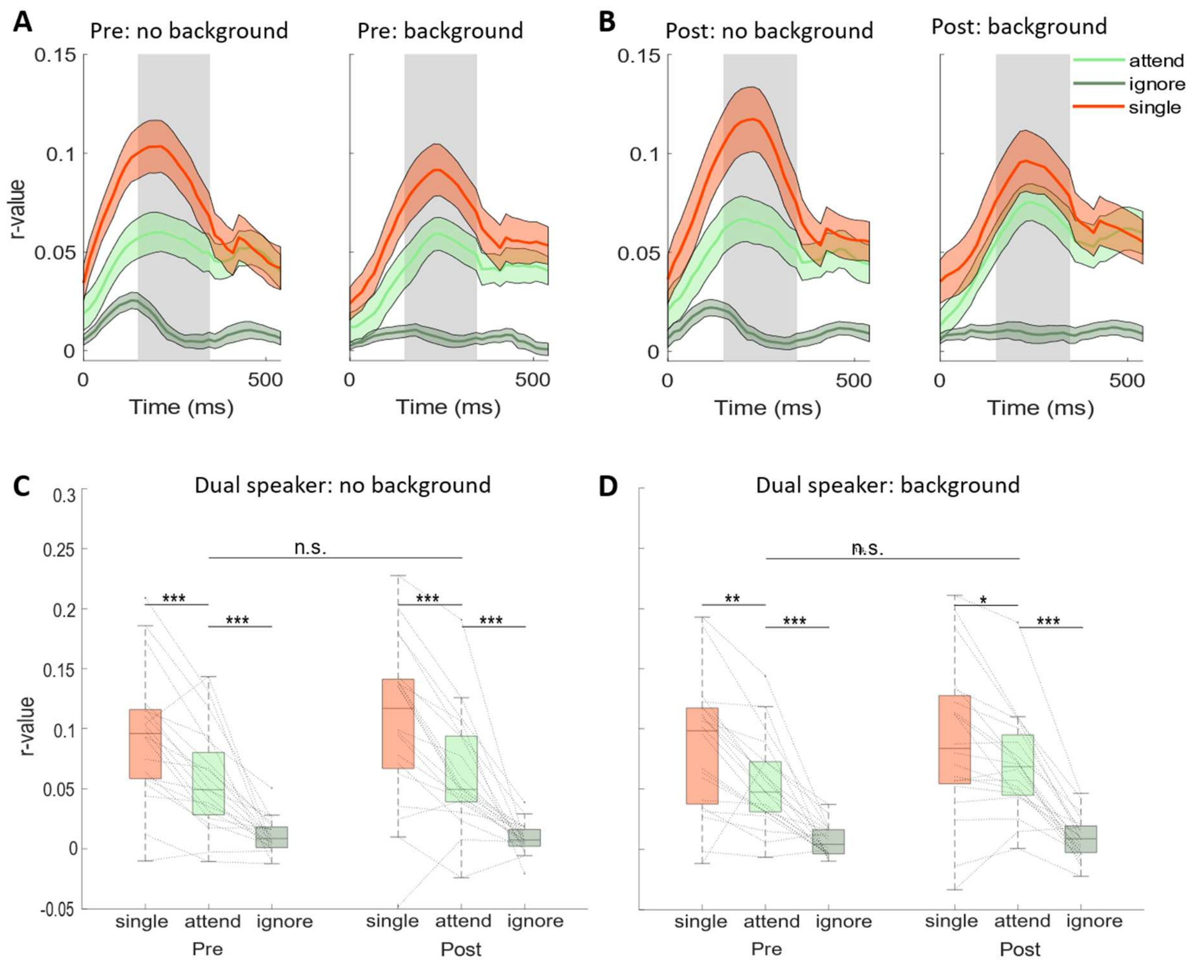

Figure 1 Subject dependent model, Lab 1 and Lab 2: A & B - Morphology of neural tracking from 0 to 500 ms relative to the speech envelope. Displayed for single (red) and dual (attended: light green, ignored: dark green) speaker conditions in Lab 1 (A) and Lab 2 (B) measurement. Shaded colored areas show +/- 1 standard error. Shaded gray area represents time window used for analysis. C & D – Comparison of neural tracking within and between Lab 1 and Lab 2 single and dual speaker conditions (C: without background cafeteria noise; D: with background cafeteria noise).

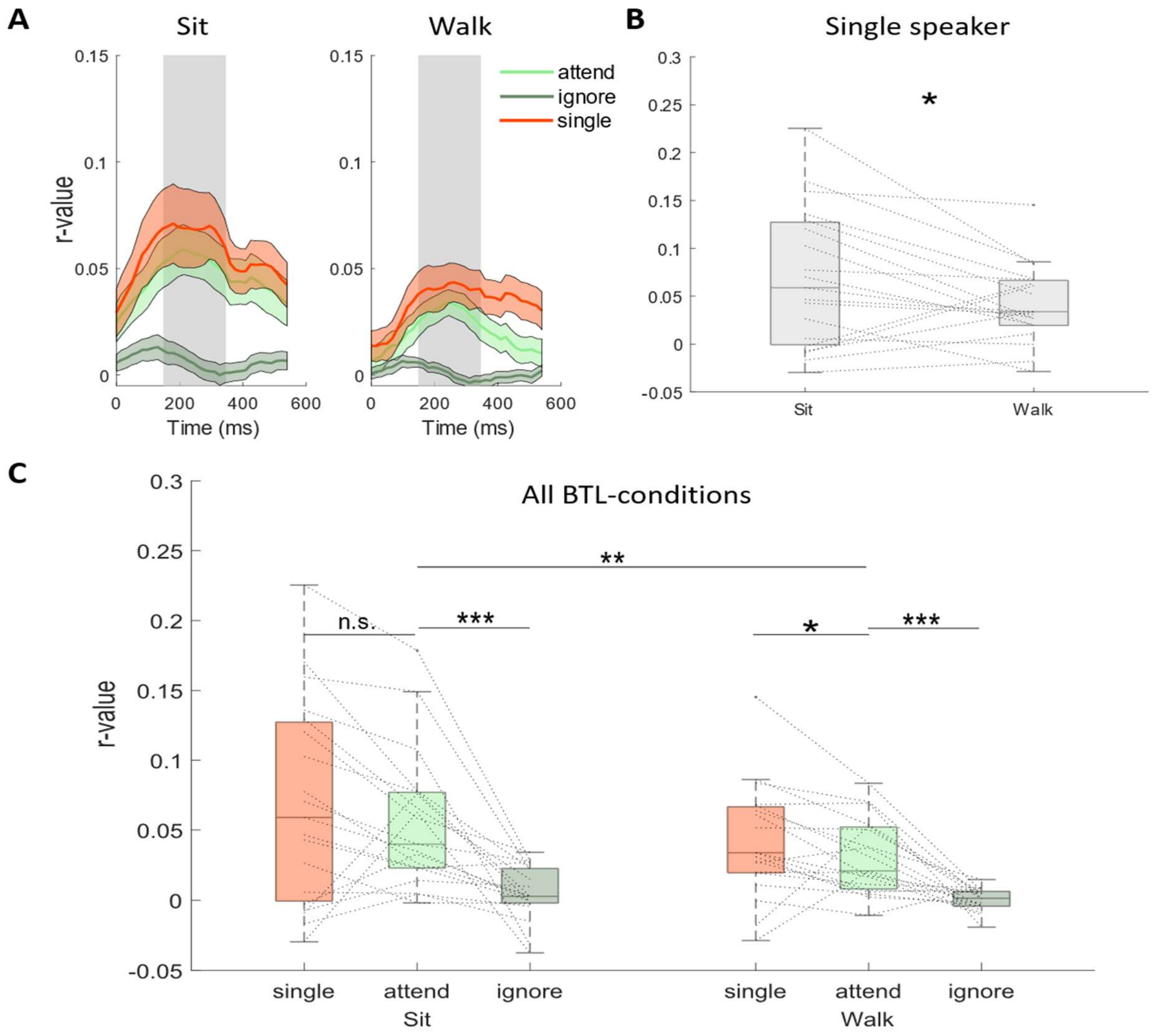

Figure 2 Subject dependent model, BTL: A – Morphology of neural tracking from 0 to 500 ms relative to the speech envelope. Displayed for single (red) and dual speaker (attended: light green, ignored: dark green) conditions in BTL movement conditions (sit and walk). Shaded gray area represents time window used for analysis. B – Comparison of single speaker conditions between BTL movement conditions. C - Comparison of single and dual speaker listening conditions within and between BTL movement conditions. Lines between boxplots show single subject statistics. Shaded colored areas show +/- 1 standard error.

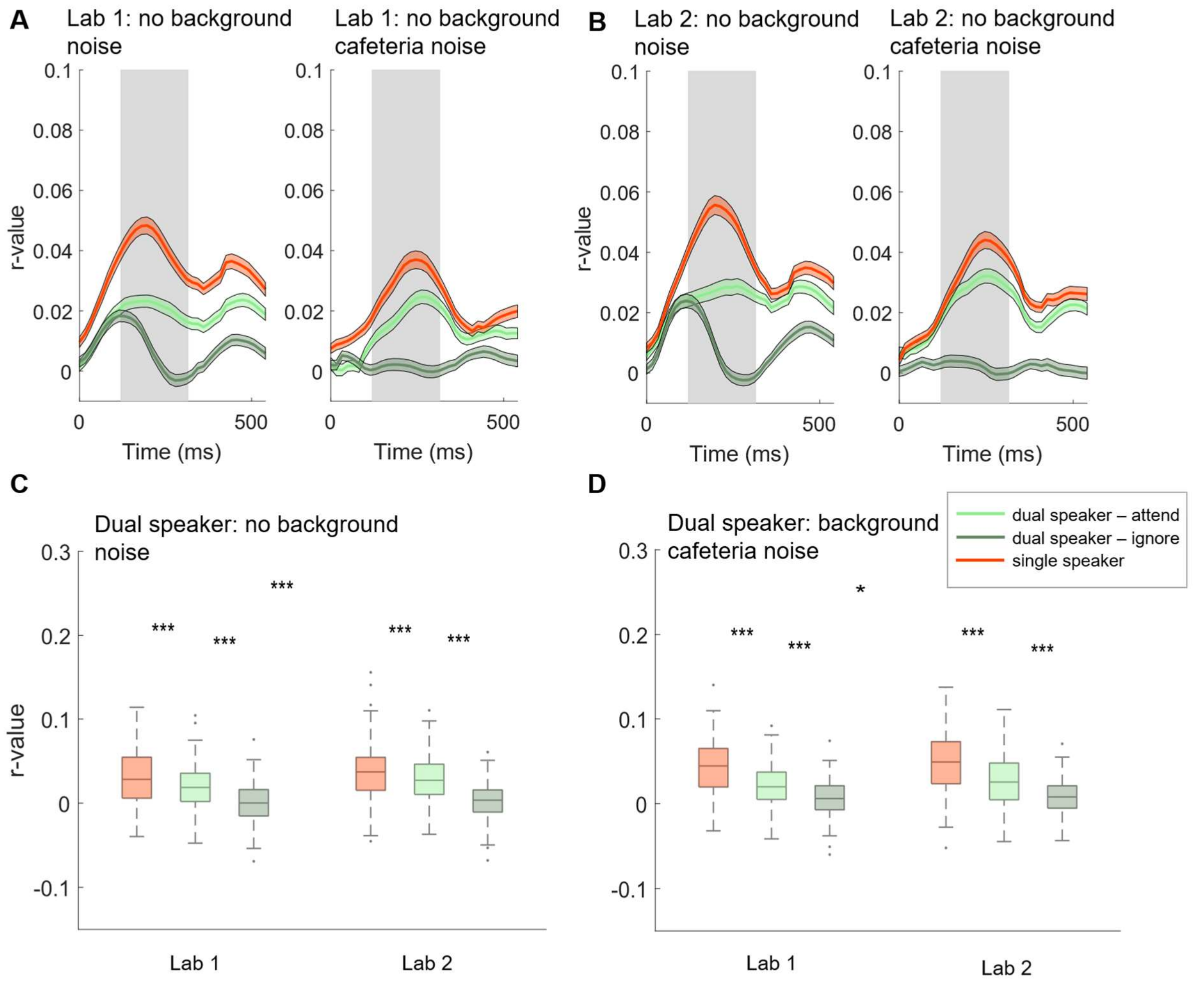

Figure 3 ICA - Subject independent model, Lab 1 and Lab 2: A & B - Morphology of neural tracking from 0 to 500 ms relative to the speech envelope. Displayed for single (red) and dual (attended: light green, ignored: dark green) speaker conditions in Lab 1 (A) and Lab 2 (B) measurement. Shaded colored areas show +/- 1 standard error. Shaded gray area represents time window used for analysis. C & D – Comparison of neural tracking within and between Lab 1 and Lab 2 single and dual speaker conditions (C: without background cafeteria noise; D: with background cafeteria noise).

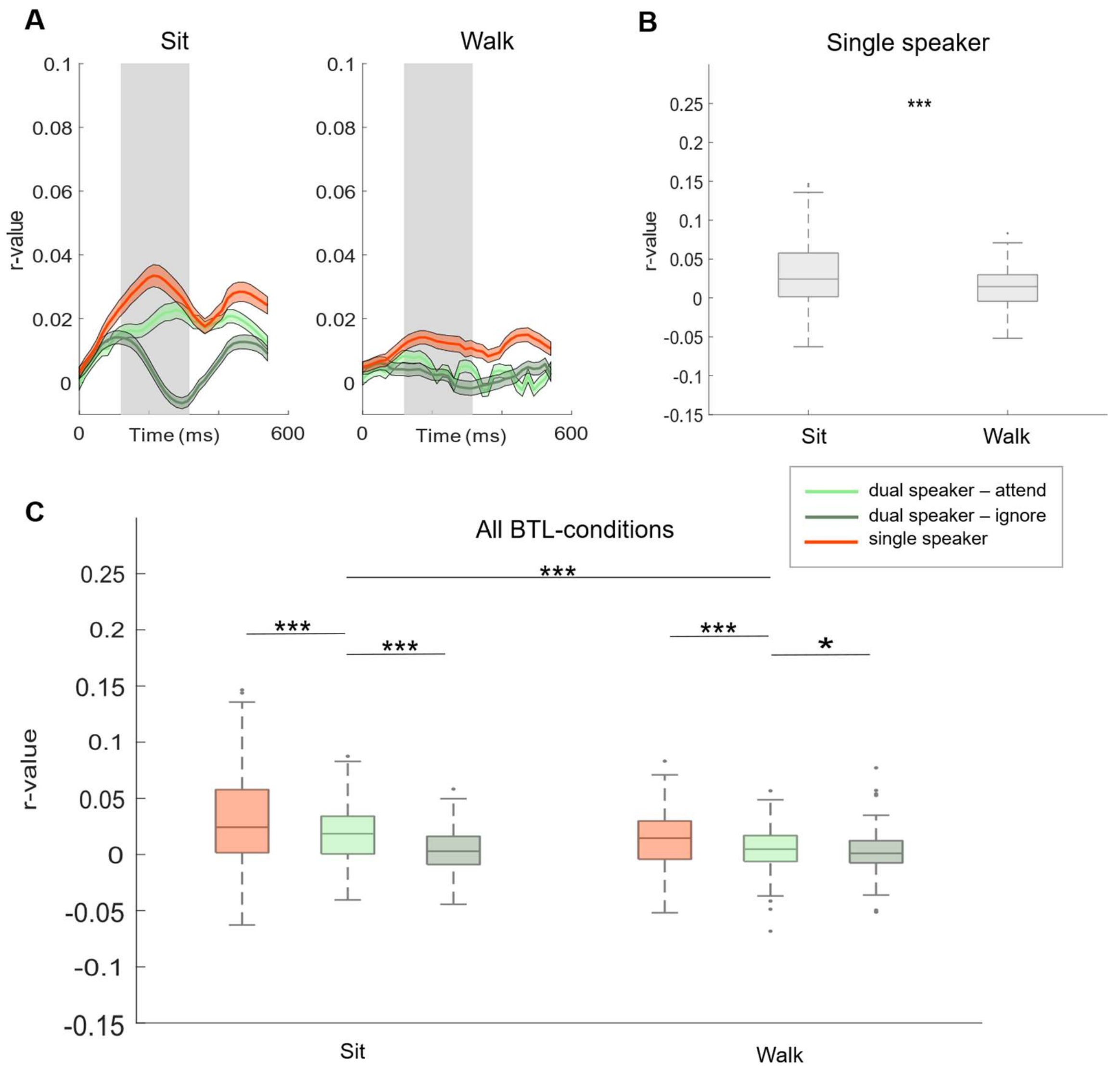

Figure 4 ICA – Subject-dependent model, BTL: A – Morphology of neural tracking from 0 to 500 ms relative to the speech envelope. Displayed for single (red) and dual speaker (attended: light green, ignored: dark green) conditions in BTL movement conditions (sit and walk). Shaded gray area represents time window used for analysis. B – Comparison of single speaker conditions between BTL movement conditions. C - Comparison of single and dual speaker listening conditions within and between BTL movement conditions. Lines between boxplots show single subject statistics. Shaded colored areas show +/- 1 standard error.
